# Double trouble: a rare case of quadricuspid aortic valve and anomalous right coronary artery

**DOI:** 10.1007/s10554-026-03669-x

**Published:** 2026-02-26

**Authors:** Aamuktha R. Karla, Malek Nayfeh, Tuna Ustunkaya, Bruno Bezerra Lima

**Affiliations:** 1 Department of Medicine, Cardiology Division, Vanderbilt University Medical Center, Nashville, TN USA; 2 Erlanger Heart and Lung Institute, University of Tennessee at Chattanooga, Chattanooga, TN USA; 3 Department of Radiology, Cardiothoracic Imaging Section, Vanderbilt University Medical Center, Nashville, TN USA; 4 Advanced Cardiac Imaging Program Vanderbilt Heart and Vascular Institute, 1215 21st Ave South, MCE5, Nashville, TN 37232 USA

**Keywords:** Anomalous Aortic Origin of Coronary Artery, Coronary Vessel Anomalies, Aortic Valve Abnormalities, Quadricuspid Aortic Valve

A 44-year-old male with high functional capacity presented with mid-sternal discomfort. Coronary CT angiography (CCTA) revealed an anomalous right coronary artery (RCA) from the left coronary cusp (Fig. 1, Panel A–B) and a quadricuspid aortic valve (QAV) (Fig. 1, Panel C). The RCA followed an interarterial course with a slit-like ostium (5.0 × 1.8 mm), acute takeoff angle, and 5.0 mm intramural segment [1].

CT-derived fractional flow reserve (FFRct) was preserved (0.95) (Panel D), concordant with normal SPECT and absence of ischemia. Although FFRct is validated mainly in atherosclerosis and may not detect dynamic compression in anomalous aortic origin, concordant normal findings supported low ischemic risk. Cardiac magnetic resonance showed mild-to-moderate aortic regurgitation (regurgitant volume 19 mL, fraction 22%), without ventricular dilation (Fig. 1, Panel E-F).

Associations between aortic valve dysplasia and anomalous coronary origin have been described [2], though quadricuspid morphology remains rare. This case highlights the complementary role of CCTA, FFRct, and guideline-supported stress testing in risk stratification and avoidance of unnecessary intervention [3] Fig. 1.

**Fig. 1:**
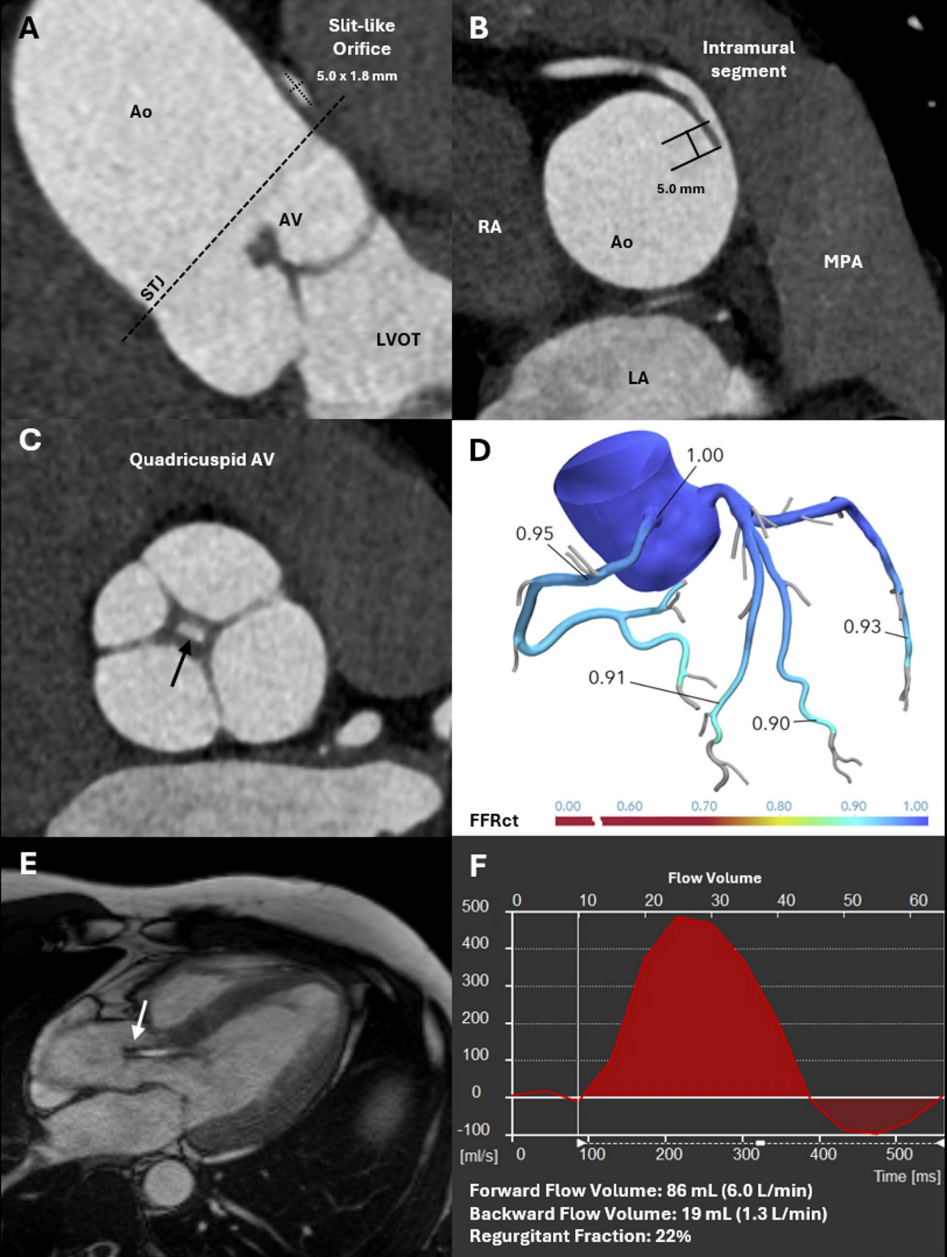
Multimodality imaging of anomalous right coronary artery and quadricuspid aortic valve with aortic regurgitation. (**A**) Coronary computed tomography angiography, CTA, demonstrating an anomalous right coronary artery, RCA, originating above the sinotubular junction with a slit-like orifice measuring 5.0 × 1.8 mm. (**B**) CTA showing a 5.0 mm intramural segment of the anomalous RCA within the aortic wall. (**C**) Short-axis CTA image of a quadricuspid aortic valve with central non-coaptation, black arrow. (**D**) Computed tomography–derived fractional flow reserve, FFRct, demonstrating preserved coronary physiology along the anomalous RCA, values ranging from 0.90 to 1.00. (**E**) Cardiac magnetic resonance imaging, three-chamber view, demonstrating an aortic regurgitation jet directed into the left ventricular outflow tract, white arrow. (**F**) Phase-contrast cardiac magnetic resonance imaging obtained above the aortic valve demonstrating mild to moderate aortic regurgitation with a regurgitant volume of 19 mL and regurgitant fraction of 22%. Abbreviations: Ao, aorta, AV, aortic valve, CTA, computed tomography angiography, FFRct, computed tomography–derived fractional flow reserve, LA, left atrium, LVOT, left ventricular outflow tract, MPA, main pulmonary artery, RA, right atrium, RCA, right coronary artery, RF, regurgitant fraction, STJ, sinotubular junction

## Data Availability

No datasets were generated or analysed during the current study.
